# Factors that provide protection against intimate partner physical violence among married adolescents in Bangladesh

**DOI:** 10.3389/fpubh.2023.1125056

**Published:** 2023-04-03

**Authors:** Mizanur Rahman, Kanta Jamil, Quamrun Nahar, Nitai Chakraborty, M. Moinuddin Haider, Shusmita Khan

**Affiliations:** ^1^Data for Impact (D4I), Carolina Population Center, University of North Carolina at Chapel Hill, Chapel Hill, NC, United States; ^2^IAP Research Inc., Dayton, OH, United States; ^3^Maternal and Child Health Division (MCHD), International Centre for Diarrhoeal Disease Research, Bangladesh (icddr,b), Dhaka, Bangladesh; ^4^Health Systems and Population Studies Division (HSPSD), International Centre for Diarrhoeal Disease Research, Bangladesh (icddr,b), Dhaka, Bangladesh

**Keywords:** intimate partner violence, intimate partner physical violence, adolescence marriage, living arrangement, spousal control

## Abstract

**Background:**

Intimate partner violence (IPV), and especially intimate partner physical violence (IPPV), perpetrated by husbands, and within adolescence marriage are pervasive in Bangladesh. Younger women are more vulnerable to IPPV.

**Objectives:**

We examined factors associated with IPPV experienced by married adolescents ages 15–19 and tested four hypotheses: (1) adolescent girls married to relatively older husbands, (2) adolescents living in extended families with parents or parents-in-law, (3) adolescents who are minimally controlled by husbands, and (4) adolescents who have a child after marriage are protective of IPPV.

**Methods:**

We analyzed IPPV data from 1,846 married girls ages 15–19 obtained from a national adolescent survey conducted in 2019–20. IPPV is defined as the respondent having physical violence perpetrated by her husband at least once in the last 12 months. We implemented logistic regression models to test our hypotheses.

**Results:**

Sixteen percent of married adolescent girls experienced IPPV. Girls living with parents-in-law or parents had adjusted odds ratio (AOR) of 0.56 (*p* < 0.001) of IPPV compared to those girls who lived with husband alone. Girls with husbands ages 21–25 years and 26 years or older had AORs of 0.45 (*p* < 0.001) and 0.33 (*p* < 0.001) of IPPV compared to those girls with their husband ages 20 and younger. Married adolescent girls who did not own a mobile phone (an indicator of spousal power dynamics) had an AOR of 1.39 (*p* < 0.05) compared to those girls who had a phone. IPPV risk increases with an increased duration of marriage for those with no living children (*p* < 0.001) but not for those with at least one living child; the risk was higher among those who had a child within the 1^st^ year of marriage than those who had not yet had a child. At a duration of 4 years and longer, IPPV risk was higher among those with no living children than those with children.

**Discussion:**

Findings related to those living with parents-in-law or parents, girls married to relatively older boys/men, having the ability to communicate with outside world, and having a child are protective of IPPV in Bangladesh are new, to our knowledge. Strictly adhering to the law that requires men waiting until the age of 21 to marry can reduce married girls' risk of IPPV. Raising girls' legal marriage age can minimize adolescents' IPPV and other health risks associated with adolescent childbearing.

## Introduction

Four types of intimate partner violence (IPV)**—**physical violence, sexual violence, stalking, and psychological aggression—are identified by the Centers for Disease Control and Prevention (1). IPV is one of the most common forms of violence against women and includes physical, sexual, and emotional abuse and controlling behaviors by an intimate partner (2). IPV is pervasive across countries, especially in those with traditional economies (2). Such violence has short- and long-term negative effects on women's health and wellbeing with varying degrees and magnitude (1). For example, morbidity and mortality of under-five children were significantly higher among women who experienced IPV than those women who did not (3, 4) and major depressive disorder was significantly higher among married adolescent IPPV victims than those who did not face IPPV (5).

Bangladesh is a country with a moderate to high level of IPV (2, 6). Younger women are more vulnerable to IPV (2, 7–11), and the country is among the top three with high incidence of adolescent marriages (Statcompiler). Six in ten women are married by age 18 and about three in ten women begin their childbearing in their teenage period (12). Child marriage or marriage before age 18 has been positively associated with IPV in Bangladesh, India, Nepal, and Pakistan (13–17).

In this paper, we examine the factors associated with IPPV experienced by married adolescents ages 15–19. We use data from a nationally representative survey of adolescents conducted in 2019–20. We concentrate on factors involving household living arrangement, partner (husband-wife) demography, partner control, and onset of childbearing after marriage. All these factors are likely to play important roles at the onset of their life-time partnership development immediately after marriage.

## Conceptual framework

**Figure 1:**
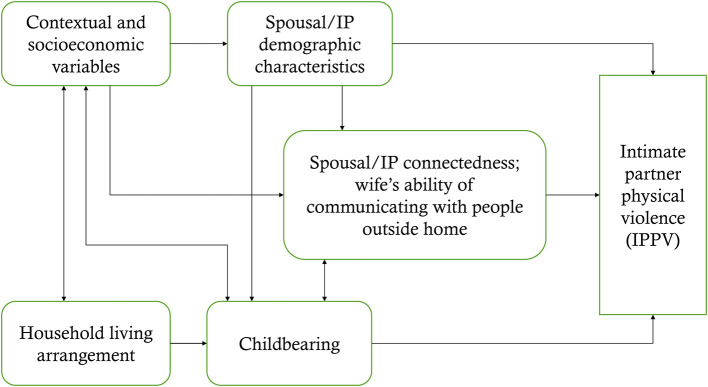
Association of spousal/intimate partners' (IP) demographic characteristics, household living arrangement, childbearing, and partner control with IPV.

In this paper, the term “intimate partner” refers to husband and wife; living together as intimate partner is uncommon and not typically allowed as a societal norm in Bangladesh. At the outset, we review the reported reasons behind IPV in Bangladesh. We then review various aspects of marriage formation processes, demography of intimate partners, living arrangement after marriage, and expectations of the newlywed female partner from the husband and other household members. We then develop hypotheses on how some selected factors can lead to intimate partner violence. A schematic diagram is shown to show the associations between factors and IPPV (Figure 1).

### Reported reasons for IPV in Bangladesh

Various reasons from more serious to day-to-day routine issues are reported as reasons behind the physical violence that are perpetrated by the husband against his wife. Lack of or inability of dowry payment (15, 18–23) and related familial conflict (24) are more serious and important factors. Other common causes of IPV include:

- A husband's controlling attitude, his sense of superiority over his wife, or his perceived and proclaimed legitimacy over his wife and related behavior (21, 25–27).
- A wife's lack of interest in sex or refusing sex at a time upon the husband's demand (28).
- An argument between a wife and her husband.
- If a wife questions her husband in day-to-day matters.
- If a wife leaves the home without letting her husband or other family member know (28–30).
- If a wife does not meet her husband's expectations in household chores (i.e., negligence of children or meals are not properly prepared) (7, 15).
- Not conforming to wearing a veil or other expected behavior (29).
- The inability to become pregnant (28).

Other family members such as in-laws (usually mother- or/and sisters-in-law) can also instigate IPV (7). However, the role of in-laws in IPV has likely minimized over time with increased education and women's greater participation in income earning (27). In Bangladesh, married women agreed that wife-beating is justified for the following reasons: argument with husband, neglecting children, going outside home without permission of husband, refusing sex when asked by husband, and burning of food (31).

### Marriage partner selection

Marriage is typically arranged by parents or grandparents, family members, and relatives (32). No or limited chance exists for potential marital partners of knowing each other although this norm is changing, especially in urban areas. Partner selection by the spouses, if any, usually occurs among relatively older people, especially those with more education. Partners are now given a chance to formally meet for a few hours immediately prior to the wedding, if desired. However, there are few cases of adolescent marriage that take place following love affair, initially without the knowledge of parents and family members and eventually family accept the couples as married.

Most couples receive gifts from the bride's family in cash, in kind, or both. Negotiated gifts from bride's family to the groom's family is known as a dowry and is a common practice, especially within low-income households. The dowry is given at the time of wedding or before, but the payment can be deferred partially or in full for months or years after the wedding.

### Partner demography

As mentioned above, teenage marriage is the cultural norm in Bangladesh, especially in rural areas, but it is still common in urban areas. Husbands are, on average, almost 8 years older than their wives (12, 32). According to the 2017–18 BDHS, 32 and 46% married women ages 15–49 had husbands 10 or more years older and 5–9 years older, respectively. Only 20% had husbands who were less than 5 years older and less than 1% had husbands their same age or younger. Data shows that the age gap between partners is gradually declining over time (Statcompiler). According to the 2019–20 BAHWS, age distribution of husbands of married adolescents was as follows: less than 10% were 20 years or younger, 46% were 21–25 years old, and 44% were older than 25 (12).

### Living arrangement after marriage

The bride moves to the household of the groom after marriage, but in rare cases the couples in Bangladesh begin their family life as a standalone, nuclear family. Some siblings living in the groom's home may be married and have children. There may be grandparents there too, along with grandparents' other married or unmarried children (married children may have their children), extending the household structure further. More than one in four families is a three-generation family according to the recent BDHS 2017–18 (12).

Some brides temporarily live with their parents, a trend increasing with the international migration of males who work in the Middle East and other countries. One in four married adolescents ages 15–19 reported that husbands live elsewhere (12). In the case of a migrant husband, the bride is likely to continue to live with her parents and her husband will visit her once every year or two. In a few cases, a groom moves to his in-laws' household, meaning the bride will live with her parents.

In the 2019–20 BAHWS, one in three married adolescent girls reported that she lives only with her husband, and not with any parents-in-law or parents (30). Married adolescent girls who live in non-nuclear households live with her husband's mother- and/or father-in-law or her own parents. Those who live with in-laws may also have sister- and/or brother in-laws in the house.

### The wife's expected role in the household

Household chores, cooking, cleaning, caring for children, and caring for older adults (if the household has such person[s]) are all carried out by the wife in Bangladesh. A husband rarely participates in these activities. The expectation is that these activities will be completed efficiently and timely. Additionally, a wife is expected to adhere to the advice and/or directions of the in-laws if she lives with them.

## Hypotheses: Partner's age, living arrangement, controlling partner, and childbearing as a cause of intimate partner violence

### Partner's age

The male partner (a husband in Bangladesh) is mainly the perpetrator of IPV, and his age is an important factor associated with that IPV. Partners of adolescents (ages 15–19) are likely to be young men (30). Youths or adolescents are likely to be impulsive in their behavior and thus are more likely to be prone to violent behavior. Relatively younger males (or youth males) may react more aggressively to situations than their relatively older counterparts merely because of the age. The United Nations defines youths as those ages 15–24.

Thus, we hypothesize that *adolescent girls who are married to youth males are more likely to encounter more violent attitudes and behavior than those who are married to males ages 25 and older*.

### Living arrangement

IPV incidence can vary within household living arrangements. The following scenarios may be considered in terms of the link between the living arrangement and IPV:

1. When an argument or other situation arises within a nuclear family (where only the spouses live), there is no mediator who can help to calm or pacify the episode and/or intervene.
2. If a couple lives with other family member(s) and an argument or an incidence that may lead to physical violence occurs, it can worsen if one or more members seem to support either of the spouses. In-laws, especially, mothers-in-law, can worsen the situation when spouses argue and this can increase the risk of IPV.
3. In contrast, there may be no incidence of IPV if a family member mediates the argument between the spouses.

Considering these scenarios, living in an extended family may be protective or harmful in terms of IPV. However, as we will see below, ideological shifts around women's roles are occurring. These are, over time, associated with social and economic transformations that lower the risk of IPV. A recent study indicates that mothers-in-law are now more tolerant about daughters-in-law, especially for those daughters-in-law who participate in income earning (33). It is therefore possible that mothers-in-law can help ease the situation and minimize the chance of IPV.

In Pakistan, support from family members was negatively associated with IPV (34). Married adolescent girls living with her parent(s) are likely to benefit from a parent moderating or intervening in an event that could lead to IPV. Schuler et al. (27) observed that with increased economic collaboration between husbands and wives, the power and importance of mothers-in-law is fast diminishing. Education is becoming more common among the current generation of brides and as husbands become more dependent on their wives' economic contributions, more egalitarian attitudes are growing.

Social isolation is a risk factor of IPV across societies (35). Living in nuclear households may increase social isolation as those who live in extended households are more likely to receive social/familial support to avoid IPV. Stoff et al. (35) found in Bangladesh that women who maintain natal family contact at least six times a year were more likely protected from psychological IPV. When they contact their natal family more than ten times a year, she is ten times more protected from the likelihood that she will be a victim of sexual IPV. Instrumental social support can also be negatively associated with psychological, sexual, and physical violence (35). This is when a bride has someone outside the home but in the village who will be willing to provide financial or other support if needed. It is also possible that community members can intervene when IPV occurs (36). Social support acted as a protective buffer against spousal physical violence (22).

In this study, we hypothesize that *a married adolescent girl living with some family members, especially parent(s)-in-law or parent(s), experiences a different risk of IPV than one living alone with her husband*.

### Controlling partner

In the Bangladesh patriarchal society, husbands have a controlling attitude toward their wives. In fact, daughters grow up under the “guardianship” of the father and through a marriage the guardianship is transferred to the husband. Wives are exposed to the risk of IPV through this controlling mechanism (21). A husband's controlling behavior is a strong predictor for domestic violence in India (37).

Partner control may be influenced by two behaviors—wife-husband connectedness and wife having a power of some kind. Wife-husband connectedness may be a condition under which the wife is less likely to be controlled by the husband if the partners are connected. A wife may be classified as connected if she feels that she enjoys spending time with husband and she talks with husband about very personal things always or most of the time. Therefore, connectedness may be treated as a proxy for controlling the wife by the husband. Negotiating as a power/skill by the wife can lessen the controlling power of the husband.

A wife may acquire empowerment from her own personality asset (e.g., negotiation) or wealth which may help lessen the controlling strength of the husband. For example, if the wife earns money through a job, she may also participate in microfinance or receive remittances from parents or relatives. This empowerment of the wife, however, may be negatively or positively associated with IPV. Women who work for money were less likely to experience IPV in Bangladesh (9, 20). Such earning is a source of empowerment which helps reduce the burden of IPV (36). Hadi (38, 39) maintained that women's productive roles through participation in credit programs and making financial contributions to their families not only improved women's positions in their households but also significantly reduced domestic violence.

However, women's participation in microfinance for earning was not associated with higher level of IPV (8). Women who meaningfully contributed to the family income through earnings were more likely to be experiencing violence (18). Women who were more equal with their husbands in their family relationships by participating in decision making increased their exposure to IPV by membership in microfinance programs (37). Women who make decisions about household purchases were more likely to experience physical abuse by their husbands (9, 40).

A relatively new idea of women's exit options from abusive marriages such as separation of marriage or divorce, which is more becoming common and recognized by the community, as indicated by Schuler and Nazneen (36). This is also a source of women's empowerment. Schuler et al. (33) observed that men's attitude of controlling their wives are changing and commented: *Men's growing acceptance of egalitarian gender norms and their self-reported decreased engagement in IPV are driven largely by pragmatic self-interest: their desire to improve their economic status and fear of negative consequences of IPV*.

Another vital source of empowerment of a married girl may be her ability to communicate with people outside home seeking advice, suggestions, or even help in crisis. Access to a mobile phone, for example, may serve this purpose, and such access may be realized through her own earnings or remittances from natal family or relatives or from her husband. This is also a source of empowerment which may help protect a wife from IPV perpetrated by her husband.

We hypothesize that *spousal control is associated with IPV, i.e., a husband who fully controls his wife is more likely to practice IPV than a husband who does not control or minimally controls. The effect of control is minimized when (a) husband and wife are connected, i.e., spouses have a sense of togetherness, and (b) wife is empowered with her ability to communicate with people outside home owing to some resources owned by her*.

### Childbearing or demonstrating the proof of fecundity

Bangladesh is an early-childbearing society, as indicated above, three in ten women begin childbearing during adolescent ages. Parents in laws, family members, and relatives encourage the newlywed couples to have a child early after marriage at whichever age the marriage takes place. It is the social norm, there is even a *pressure* for newlyweds in favor of early childbearing and proving their ability of childbearing (41). Young male adults working abroad is common in Bangladesh, and Gipson and Hindin (41) observed in the communities with male migration observed that having a child may be one way to cement the bond between the husband and wife before he leaves for the country for work, meaning the encouragement of childbearing immediately after marriage.

Many young newly married girls/women fear that they may become childless or may not be able to bear a child with passage of time which is bolstered by their natal and marital family members (32). The family wants to see that the wife has given birth to a child. This has probably been derived from the perception that exists in subsistence and agricultural economies that children are beneficial to family earning and that children can join the workforce at an early age. It is also expected that a son will be born and will reach adulthood before the father reaches old age or dies so the son can take of the family.

Pressures come from in-laws to have a child immediately after marriage in India (42). The decision to have a child after marriage is largely influenced by in-laws and husbands—a woman's choice of low importance in Bangladesh (43). Delaying the first birth after marriage can cause rumors of infertility, bring shame upon the family, and in some cases lead the husband's family to seek another wife for their son (44). Additionally, social stigma for childless women, emigration of husbands, and the belief that using modern contraceptives prior to the birth of the first child results in infertility also inhibits couples from delaying their first pregnancy. It should be mentioned that there has been a rapid demographic transition in Bangladesh wherein fertility declined from over 6.0 births per woman to 2.3 births and life expectancy has declined from about 50 years to over 70 years in the last 40 years, the perceived benefit of early childbearing seems to remain in the older generation.

The young newly married women, e.g., adolescents ages 15–19 want to delay their childbearing and the family members encourage or sometimes pressurize newly married ones to have a child earliest possible time. While the women may pursue the delay, her spouse may support family members' view of early childbearing and demand the wife to have a child, and if there is disagreement the husband may become violent. In India, IPV was associated with the intention of delaying the first birth (45).

Therefore, we hypothesize that *delaying of childbearing is associated with IPV among adolescents in Bangladesh*.

## Methods and procedures

### Data

Data for this study come from the nationally representative Bangladesh Adolescent Health and Wellbeing Survey 2019–20 (30) [The survey details are given in NIPORT (30), including the questionnaires.]. The age limit for adolescents in this survey was 15–19 years. The survey was based on a two-stage stratified sample of households, which involved sampling of primary sampling units (PSU), and sampling of households. At the first stage, PSUs were randomly selected from each stratum according to probability proportional to size of the number of households. PSUs were randomly and equally divided into Type 1 and Type 2 PSUs, and information on physical violence was collected from Type 2 PSUs. At the second stage, 67,093 households were selected from where 7,800 unmarried female adolescents, 4,926 ever-married female adolescents (2,904 in Type 1 and 2,022 in Type 2), and 5,523 unmarried male adolescents were selected for interview. The response rate for married adolescent sample was 97.2%. Data were collected on paper and pencil through face-to-face interviews at the home of the respondents by using a structured questionnaire. Our sample come from Type 2 PSUs and thus Type 2 questionnaire, and the respondents were 2,022 married adolescents.

All target adolescents in the selected households were included in the survey. However, if there were more than one adolescent in a household, the module that has questions on violence was not implemented. This was done on a consideration that multiple sample adolescents from the same household may feel embarrassed of the reportable incident(s) that occurred in the past 12 months with a fear that other respondent(s) would know about it. Only 3.3% of sample households had multiple adolescents. For our analysis we excluded 200 married adolescents whose husband resided outside home and did not visit home in past 12 months.

Our analysis considers appropriate sampling weights calculated based on the complex sampling design of the BAHWS 2019–20, and our sample consisted of 1,822 (weighted *n* = 1,846) married adolescent girls ages 15–19.

### Study variables

Table 1 shows the variables and measurements of *factors* and *outcome* used in this analysis.

**Table 1:** Indicators and measurement of factors and outcome.

**Factor/outcome**	**Indicator**
Intimate partner physical violence (IPPV)	Experience of physical violence perpetrated by the husband or husband along with other family member •Those who reported having *experienced* physical violence at least once in the 12 months preceding the survey, i.e.: •Slapped, pushed, or pulled hair • Punched, thrown something at her, or hit with a stick or something heavy • Kicked, dragged, or beat up • Tried to choke her or burn her on purpose with something hot (fire, object, acid) •Threatened or attacked her with a knife, gun, or any other weapon
Socioeconomic	•Years of schooling (≤ 5, 6–9, 10+) •Household asset quintile (Bottom 40%, Middle 20%, and Upper 40%)^*^ •Residence (Urban, Rural) •Region of the country (Western [Rangpur, Rajshahi, and Khulna Divisions], Central [Mymensingh, Dhaka, and Barishal Divisions], Eastern [Sylhet and Chattogram Divisions]).
Attitude toward gender roles	•Egalitarian if *disagree* to each of the four statements: (a) It is important that sons have more education than daughters; (b) outdoor sports are only for boys not for girls; (c) household chores are for women only, not for men, even if the woman works outside the house; and (d) women should not be allowed to work outside of home. • Inegalitarian, otherwise.
Attitude toward gender responsibilities and spousal/IP power dynamics	•Egalitarian if *disagree* to each of the three statements: (e) Looking after the household and kids is the responsibility of women only; (f) a woman should always listen to her husband even if she disagrees; and (g) a husband has the right to physically assault or beat his wife if she does not listen to him. • Inegalitarian, otherwise.
Spousal/IP demographic characteristics	•Husband's age ( ≤ 20, 21–25, and 26+) • Duration of marriage (0–1 year, 2–3 years, and 4+ years)
Household living arrangement	Living arrangement •With husband and children (if any) •With husband, parents in laws or parents, and children (if any) •Husband lives elsewhere but she lives with (a) parents in laws or (b) parents, or (c) lives alone. (There may be child[ren] if there are any). In group (c), there may be person(s) of other type of relationship(s).
Spousal/IP connectedness	Connectedness with husband •*Connected* (wife enjoys spending time with husband and talks with husband about very personal things most of the time or always) •*Weakly connected*, otherwise
Wife's ability of communicating with people outside home	•Having her own mobile phone (yes, no)
Childbearing	•Having living child(ren) (0, 1+)

* Household asset quintile is constructed by the household ownership of a set of assets as reported by the respondent or observed by the interviewer at the survey.

#### Intimate partner physical violence

The 2019–20 BAHWS had five questions on the nature of *physical violence* experienced by married adolescents, see the first row in Table 1. If such violence was perpetrated by her husband at least once in the past 12 months of the survey is treated as IPPV. Answering *Yes* to at least one of the questions is defined as an outcome of IPPV and coded as 1, and otherwise as 0.

#### Spousal demographic characteristics

Husband's age is categorized as ≤ 20, 21–25, and 26+ and ≤ 20 category is treated as the reference category in the logistic regression. Duration of marriage is divided in to three groups: ≤ 1 year, 2–3 years, and 4+ years and the 1^st^ year of marriage is treated as reference category.

#### Living arrangements

We consider three categories of living arrangements: (a) with husband (and children, if any), (b) with husband and parents-in-law or parents and children if any, and (c) husband lives elsewhere but wife lives with parents-in-law or parents and children, if any. In group (c), there may be person(s) of other type of relationship(s). The category (a) here is treated as the reference category in the logistics regression.

#### Spousal control

We use two variables to capture the effect of spousal control: respondent's connectedness with her husband and respondent having her own mobile phone. On the married girl's connectedness with her husband, we assume that a married girl who has a feeling of connectedness with her husband, she is less likely to be fully controlled by her husband. A wife possessing a phone and using it is likely due to indicate two characteristics: (1) she can communicate with people outside home and (2) she has power, economic or otherwise, acquired either by her own earning, through inheritance of resources from relatives, or through negotiation with the husband. Such characteristics can soften or minimize a husband's controlling attitude and/or actual control over his wife.

#### Childbearing

The childbearing indicator is dichotomized as having at least one living child at the time of survey (coded as 1) and no living child is coded as zero.

We also include the following variables (see Table 1 for definitions):

- Years of schooling in three categories— ≤ 5 years (reference category), 6–9 years, and 10+ years.
- Attitude toward gender roles—Inegalitarian (reference category) and egalitarian.
- Attitude toward gender responsibilities and power dynamics in the family—Inegalitarian (reference category) and egalitarian.
- Household asset quintiles—Bottom 40% (reference category), middle 20%, and upper 40%.
- Region of the country—Western (reference category), Central, and Eastern.
- Residence—Urban (reference category) and Rural.

It is possible that IPPV is influenced by husband's education, husband's smoking or drinking habits, or respondent's parental education but the BAHWS 2019–20 did not collect information on those, neither did we have adolescents' information on religion.

### Statistical analysis

We estimate the effects of various variables on IPPV in logistic regression model. The independent variables included in the regression model to represent the factors indicated in the conceptual framework. We did not use stepwise procedure to include or exclude the variables. We checked for multicollinearity between independent variables and none of the tests required to exclude any independent variables. For example, the mean Variance Inflation Factor (VIF) was 1.3 (and maximum limit was 1.76) which is much less than 10, the acceptable limit. We used STATA version 17. There were only seven records (0.38%) with missing values in two variables, and the STATA exclude those records that has a missing value.

## Results

### Sample characteristics

Table 2 shows the distribution of 1,846 married adolescent girls according to categories of factors considered in the analysis. About one in four is an urban resident; about two in five are each from Western and Central regions; and about two in five from the upper 40% and about one in three from the bottom 40%. About one in four had no or less than 6 years of schooling, about half had 6 to 9 years of schooling, and about one in four had ten or more years of schooling. About two in five married girls had egalitarian attitudes toward gender roles (see Table 1) and about one in three had egalitarian attitudes toward gender responsibilities and spousal power dynamics.

**Table 2:** Description of sample of currently married adolescents ages 15–19 who are exposed to IPPV, Bangladesh Adolescents Health and Wellbeing Survey 2019–20.

	**Sample size (n)**	**% Sample**	**% Experienced IPPV at least once in last 12 months**	**Chi-square**	***P* value**
Total	1,846	100.0	16.0		–
**Residence**				0.65	0.45
Urban	440	24.9	14.7		
Rural	1,405	76.1	16.4		
**Region**				4.04	0.18
Western	777	42.1	16.8		
Central	726	39.3	16.7		
Eastern	343	18.6	12.3		
**Household asset quintile**				16.92	0.0007^***^
Bottom 40%	803	43.5	19.9		
Middle 20%	408	22.1	14.1		
Upper 40%	635	34.4	12.1		
**Years of schooling**				37.86	0.0000^***^
0–5 years	428	23.1	22.4		
6–9 years	974	52.8	17.0		
10+ years	444	24.1	7.4		
**Attitude toward gender roles**				7.69	0.017^*^
Inegalitarian	1,135	61.6	17.9		
Egalitarian	706	38.4	13.0		
**Attitude toward gender responsibilities and spousal power dynamics**				1.38	0.286
Inegalitarian	1,256	68.2	16.6		
Egalitarian	586	31.8	14.4		
**Household living arrangement**				35.72	0.000^***^
With husband (and children, if any)	460	24.9	24.5		
With husband, parents-in-law, parents (and children, if any)	1,176	63.7	13.8		
Husband lives elsewhere but she lives with parents in laws, parents, or lives alone (and children, if any)	209	11.3	9.0		
**Husband's age**				22.04	0.000^***^
≤ 20 years	172	9.3	26.1		
21–25 years	856	46.4	17.3		
26+ years	817	44.2	12.4		
**Marriage duration**				30.88	0.000^***^
0–1 year	892	48.3	11.1		
2–3 years	617	33.4	21.1		
4+ years	337	18.3	19.5		
**Connected with husband**				6.15	0.012^*^
Connected	844	45.7	13.7		
Weakly connected	1,001	54.2	17.9		
**Owning a phone**				12.06	0.000^***^
Having a mobile phone	916	49.6	12.9		
Having no mobile phone	930	50.4	18.9		
**Childbearing**				25.02	0.000^***^
Having no living children	1,039	56.3	12.2		
Having at least 1 child	805	43.6	20.8		

* *p* < 0.05;

*** *p* < 0.001.

About one in four married girls lived alone with husband (including own children if any), about two in three lived in extended family with husband and at least one parent or parent-in-law, and about one in ten lived without husband but with at least one parent or parent-in-law.

While the girls were ages 15–19, 9, 46, and 44% of their husband were 20 or below, 21–25, and 26 and higher years of age, respectively (Table 2). Nearly half (48%) were married for less than 2 years, one third (33%) for 2–3 years, and about one in five (18%) were married for 4 or more years.

Slightly less than half of married girls were “connected” with their husband, measured by the indicator “enjoy spending time with husband and can talk with husband about very personal things most of the time or always” (Tables 1, 2). Also, half of the girls had their own mobile phone.

Regarding childbearing, 44% of married girls had at least one surviving child (Table 2).

### Prevalence and pattern of IPPV: Bivariate findings

Table 2 (Column 4) shows the prevalence of IPPV according to their characteristics. Sixteen percent of married girls reported that they experienced IPPV at least once in the past 12 months. IPPV was similar for rural and urban areas; it was slightly lower, not significantly, in the Eastern region than other regions; about 20% girls in the bottom 40% households experienced IPPV compared to 12% in the upper 40% households, the difference is significant (*p* < 0.001). IPPV substantially decreases with education, from 22% among girls with no or below 6 years of schooling to 17% among girls with 6–9 years of schooling to only 7% among those with 10 or more years of schooling (*p* < 0.001).

Girls with inegalitarian attitude toward gender roles had an IPPV rate of 18% compared to 13% among egalitarian (*p* < 0.05). IPPV differential in terms of attitude toward gender responsibilities and spousal power dynamics was similar between the inegalitarian and egalitarian groups (17 vs. 14%, not significant).

IPPV is strongly associated with living arrangement (*p* < 0.001); 25% girls living with husband alone (i.e., nuclear family) experienced IPPV compared to 14% among those who lived with husband and parents-in-law, or parents. Those girls who lived without her husband (i.e., her husband lived elsewhere but visited home sometimes) but with in-laws or parents have significantly lower IPPV rate of 9% than other groups.

IPPV risk significantly decreases from 26% among those whose husband was 20 years old or younger to 17% among those whose husband were 21–25 years old to only 12% among those with husband of 26 years or older (*p* < 0.001). The risk increases with the duration of marriage (11% among those with 0–1 year of duration vs. about 20% among those with higher duration [*p* < 0.001]).

IPPV risk is moderately associated with girls' “connectedness” with their husbands (*p* < 0.05)−14 vs. 18% for the connected and weakly connected groups, respectively. Not having a mobile phone exposes girls to IPPV as it was 19% among those who did not have a phone compared to 13% among those who had a phone (*p* < 0.001).

IPPV rate was 21% among married girls without a living child compared to only 12% among those who had at least one living child (*P* < 0.001).

### IPPV risks: Multivariate findings

We modeled IPPV risk associated with factors under consideration by using logistic regression (Table 3). We considered contextual and socioeconomic factors such as rural-urban residence, geographical region, household asset quintiles, girls' education, and two variables capturing girls' attitudes toward gender roles. The model includes spousal demographic characteristics (husband age and marital duration), spousal connectedness and the girl's ownership of a mobile phone, household living arrangement, and a childbearing (measured as having at least one living child) indicator. We included the interaction term “living children x marriage duration.” Adjusted odds ratios (AOR) are shown in Table 3.

**Table 3:** Binary logistic regression-based unadjusted odds ratios (OR) and adjusted odds ratios (AOR) of IPPV among currently married adolescents ages 15–19, Bangladesh Adolescents Health and Wellbeing Survey 2019–20.

	**Bivariate**		**Multivariate**	
**Factors**	**OR**	**95% confidence interval**	**AOR**	**95% confidence interval**
**Residence**				
Urban	1.00		1.00	
Rural	1.13	(0.80, 1.61)	1.04	(0.72, 1.50)
**Region**				
Western	1.00		1.00	
Central	0.99	(0.75, 1.32)	1.11	(0.82, 1.51)
Eastern	0.70	(0.44, 1.10)	0.83	(0.53, 1.30)
**Household asset quintile**				
Bottom 40%	1.00		1.00	
Middle 20%	0.66^*^	(0.47, 0.94)	0.81	(0.55, 1.18)
Upper 40%	0.56^***^	(0.41, 0.77)	0.82	(0.57, 1.19)
**Years of schooling**				
0–5 years	1.00		1.00	
6–9 years	0.71^*^	(0.52, 0.97)	0.86	(0.62., 1.20)
10+ years	0.28^***^	(0.18, 0.43)	0.47^**^	(0.29, 0.77)
**Attitude toward gender roles**				
Inegalitarian	1.00		1.00	
Egalitarian	0.68^**^	(0.51, 0.91)	0.83	(0.59, 1.18)
**Attitude toward gender responsibilities and spousal power dynamics**				
Inegalitarian	1.00		1.00	
Egalitarian	0.85	(0.63, 1.14)	1.27	(0.88, 1.83)
**Husband's age**				
≤ 20 years	1.00		1.00	
21–25 years	0.59^*^	(0.39, 0.90)	0.45^***^	(0.29, 0.70)
26+ years	0.40^***^	(0.26, 0.62)	0.33^***^	(0.20, 0.52)
**Marriage duration**				
0–1 year	1.00		1.00	
2–3 years	2.15^***^	(1.58, 2.92)	1.75^*^	(1.10, 2.80)
4+ years	1.95^***^	(1.35, 2.81)	3.72^**^	(1.65, 8.37)
**Connectedness with husband**				
Connected	1.00		1.00	
Weakly connected	1.38^*^	(1.05, 1.82)	1.30	(0.98, 1.72)
**Owning a mobile phone**				
Having a mobile phone	1.00		1.00	
Having no mobile phone	1.57^**^	(1.19, 2.06)	1.39^*^	(1.03, 1.89)
**Household living arrangement**				
With husband (and children, if any)	1.00		1.00	
With husband, parents-in-law, parents (and children, if any)	0.50^***^	(0.37, 0.67)	0.56^**^	(0.42, 0.78)
Husband lives elsewhere but she lives with parents in laws, parents, or lives alone (and children, if any)	0.31^***^	(0.18, 0.53)	0.44^**^	(0.25, 0.79)
**Childbearing**				
Having no living children	1.00		1.00	
Having at least 1 child	1.90^***^	(1.45, 2.49)	1.90^*^	(1.07, 3.39)
**Living children** × **Marriage duration**				
Having a child × Marriage duration (0–1 year)			1.00	
Having a child × Marriage duration (2–3 years)			0.77	(0.37, 1.60)
Having a child × Marriage duration (4+ years)			0.26^**^	(0.09, 0.70)

* *p* < 0.05;

** *p* < 0.01;

*** *p* < 0.001.

#### Partner's age

AOR of IPPV is 0.45 and 0.33, respectively, for girls whose husband were ages 21–25 and 26 or more years old compared to girls with husband younger than 21 (*p* < 0.001) (Table 3). These findings demonstrate that girls with husbands ages 21–25 and 26 or higher have 55% ([1.00–0.45] × 100) and 67% ([1.00–0.33] × 100) lower odds of IPV than their counterpart girls with husbands younger than 21. Thus, we find that adolescent girls marrying relatively older husband provide a IPPV protective effect, and our hypothesis is supported.

#### Living arrangements

Table 3 shows that adolescent girls who lived with their parents-in-law or parents have an AOR of 0.56 (*p* < 0.01), meaning that the odds of their IPPV are 44% lower than those girls who live with her husband alone or within a nuclear family. Thus, living with in-laws or parents provide a protective effect on IPPV, and a support for our hypothesis. Girls who live with in-laws or parents, but without their husbands, have even lower odds of IPPV, i.e., 56% ([1.00–0.44] × 100) lower than girls who live with alone with their husbands or in nuclear families. These lower odds reflect the effect of infrequent exposure to IPPV as the husband lived elsewhere and visited the wife and family occasionally.

#### Spousal control

Spousal connectedness is not associated with IPPV as indicated by the non-significant AOR related to the category “weakly connected,” although it was significant in the bivariate association. Owning a mobile phone is significantly associated with the risk of experiencing IPPV as AOR of not owning one is 1.39 and is statistically significant (*p* < 0.05).

#### Childbearing

Our measure of childbearing is whether a girl has at least one living child (Table 2). Our hypothesis is that a girl who survives having a child is less likely to experience IPPV risk than her counterpart without a child. Having a living child is associated with marriage duration which is associated with IPPV as shown in bivariate findings (Table 2). We include an interaction between marriage duration and the childbearing indicator in Table 3.

**Figure 2:**
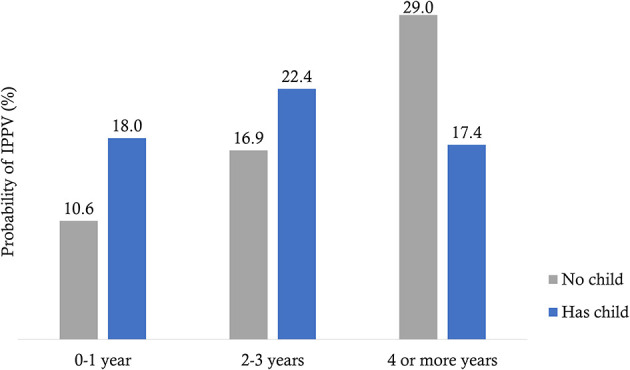
Predicted probability of IPPV based on model interaction between marriage duration and having a child.

Significant interaction occurs between having a child and the duration of marriage (Table 3). Figure 2 shows three distinct effects:

- IPPV risk for girls who did not a have living child increases from 11% during year 0–1 to 17% during years 2–3 to 29% after 4 or more years of marriage.
- IPPV risk is higher among those who have a child immediately after marriage (0–1 year) than those who have not yet have a child (18 vs. 11%).
- IPPV risk for girls with a child is about similar for the years of marriage duration.

We found that the longer girls wait to have a child the higher the risk of IPPV, and thus, our hypothesis is supported.

### Effects of other factors

Rural-urban residence and region were not significantly associated with IPPV in the bivariate analysis (Table 2) nor in the multivariate regression (Table 3). Household asset quintiles and girls' years of schooling were significantly associated with IPPV in bivariate analysis but only years of schooling is significantly associated with IPPV in the multivariate regression (*p* < 0.01). IPPV risk is significantly lower among girls who have 10 or more years of schooling than others. Among the two factors influencing attitudes toward gender roles, none were significantly associated with IPPV according to the regression models.

## Discussion

We analyzed intimate partner physical violence data from 1,846 married adolescent girls ages 15–19 based on the nationally representative adolescents survey conducted in 2019–20. We tested four hypotheses—adolescent girls married to husbands ages 25 and older, adolescents living in extended families with parents-in-law or parents, adolescents who are minimally controlled by their husbands, and adolescents who have a child immediately after marriage are protective of IPPV in Bangladesh. We find support in favor of each of the hypotheses. The findings are new, to our knowledge, except for that on the association between spousal control and IPPV.

The finding that girls married to husbands beyond the age of youthhood, i.e., 25 years or older, are protective of IPPV is like that of Adebowale (46) in Nigeria which indicates that women married to relatively older husband (measured by age difference between husband and wife) have lower likelihood of IPV in general and IPPV and emotional violence, but not true for sexual violence. Our finding implies that strictly adhering to the Bangladesh law that requires men marrying at 21 or later can reduce married girls' risk of IPPV. Increase in males' ages at marriage will benefit adolescent girls from the risk of IPV as well as through their reduced likelihood of adolescence marriage, and men should be encouraged to marry girls who are 20 or older or at least 18 years old girls as required by law.

We find that married adolescents living with parents-in-law or parent(s) helps reduce IPPV. Various studies and anecdotes indicate that living with in-laws, especially mother-in-law, is a risk of IPPV, but Schuler et al. (33) observed that there has been ideological change among in-laws wherein they believe that daughters-in-law need to be treated with dignity and respect. This is because of social and economic development and their daughter-in-law's increased economic role in families. Our finding is consistent with the notion of changed attitudes toward compromised behavior of husbands in terms of IPPV.

As Bangladesh is in transition from a traditional to contemporary economy, it is still expected that newlywed couples will begin their lives in extended family settings and then move toward nuclear ones. Our findings show that 25% married adolescents live in nuclear families, 64% with parents-in-law or parent(s), and 11% with parents-in-law or parent(s) but the husband lives elsewhere (usually for work). One reason young couples live in a nuclear family is when one of the spouses' (mostly the husband's) workplace is distant from the husband's natal home. Some young girls work in the non-agricultural sector (e.g., garment factories) and their husbands may accompany her to live in city areas. The proportion of this nuclear living arrangement is probably increasing because of the growing economic activities of the non-agricultural sector, and thus, likely increases IPPV risk for young married women.

A husband's control of his wife is a source of IPPV, but this control is moderated or lessened when his wife has some power. This can mean having a phone that enables her to communicate with the outside world. Owning a phone may also be related to her economic power, helping reduce the burden of IPPV. We argue that ownership of a mobile phone is possible through a wife's income earnings or through remittance obtained from relatives or even from her husband. However, the literature suggests, as we show in our conceptual framework, that income earning can have both negative and positive effect on IPV in Bangladesh (18, 36, 38–40). The negative association of having a phone and IPPV is likely to work through social isolation and IPV hypothesis as well as instrumental social support and IPV hypothesis (35). The social isolation hypothesis asserts that those women who communicate with natal or other kins or relatives more frequently are less likely to experience IPV and the instrumental social support hypothesis claims that those women who have someone in the same or neighboring communities willing to provide support are less likely to face IPV. In Bangladesh, instrumental support is available even at the local government level through the newly enacted Domestic Violence (Prevention and Protection) Act 2020 (7). Phone calls can be a vehicle of communication to satisfy these hypotheses and thus likely reduce IPPV. IPPV is likely to decline over time with economic growth, which is occurring rapidly in Bangladesh, that benefits women in owning mobile phone.

We did not find any significant association between spousal connectedness and IPPV. Our argument was that connectedness between spouses would reduce the feeling of husband's control over his wife and thus lower IPPV. One reason why we did not find significant association between connectedness and IPPV may be that the indicator we used is not an appropriate measure of connectedness. It is also possible that IPPV itself has a negative effect on connectedness in that a wife experiencing IPPV does not feel close to her husband. Without closeness, a wife may not share personal ideas with her husband, and thusly there is no significant association between connectedness and IPPV.

Childbearing is strongly associated with IPPV, specifically in that IPPV continues to increase with marriage duration if the wife does not have a living child. In contrast, having a living child puts a married girl at a moderate risk of IPPV in the early years of marriage but it does not increase with marriage duration. This finding is consistent with the common belief that the family, including the in-laws, and the society in general expect that a child should be born immediately after marriage as observed by previous researchers (41, 42). If that does not happen the married girl is at the risk for IPPV.

This finding is disturbing because childbearing during adolescence is harmful for both maternal and infant/child health. Healthcare providers recommend avoiding having a birth before age 20. Adolescent girls are in a quandary and those who desire to follow the health advice intend to use contraception. But pressures come from in-laws and family against contraceptive use at this time of her life, and, in some cases the husband joins the family against contraceptive use. This situation may lead to violence against the girl.

We are in a dilemma; should we recommend delaying childbearing after age 20 as recommended by public health practitioners? If we do, the adherent girls will be at increased risk of IPPV. Also, a significant portion of married adolescent girls who had a birth reported that they wanted to have the birth later, as shown in our companion study (5). Adolescent girls who wanted to have the birth later had 1.30 times higher risk of major depressive disorder than those who wanted the birth earlier.

We will still recommend waiting to have children after the age of 20. Following our recommendation of raising the age at marriage, we reiterate that an increase in age at marriage will minimize or even eliminate the chance of adolescent childbearing and thus reduce maternal and child health risks and the risk of IPV. However, there is only a slow increase in age at marriage—it has increased from 15.3 years in 1993 to 17.5 years in 2017, roughly a year per decade. A germane question is how can we accelerate the pace of increasing age at marriage? An obvious solution to this is keeping girls in school for 12 years of education. Although there has been a praiseworthy improvement in education in Bangladesh, only 34%, or one in three, married women aged 20–24 had 10 or more years of schooling in 2017–18. In many countries it is required that young people complete 12 years of schooling (i.e., high school graduation) and by that time they are usually 18 years old or over. Providing girls with high school or higher education also helps nation building and thus facilitating social and economic development. Girls from families that cannot afford this may be given scholarships. Cost-benefit analysis will likely show this is a noble option, and future research should be conducted in this area.

An important limitation of this study is that we analyzed cross-sectional IPPV data of quantitative nature from an adolescent health and wellbeing survey which did not have more in-depth information on issues related to IPPV. We only identified factors affecting IPPV but do not know the exact mechanisms of how these factors caused IPPV. We exercise caution that we measure association but not cause-and-effect relationships between various factors and IPPV. Further research particularly of qualitative nature can help better understand such mechanisms of IPPV among married adolescents. There is dearth of qualitative studies of IPV in Bangladesh, e.g., 6 in his review mentioned that there were 17 quantitative studies, 11 jointly quantitative and qualitative, and only 3 qualitative studies.

To conclude, married adolescent girls living with parents-in-law or parents, girls married to relatively older boys/men, having a power of communicating with outside world, and having a child immediately after marriage are protective of IPPV in Bangladesh. Strictly adhering to the law that requires men to marry at 21 or older can reduce married girls' risk of IPPV. Raising girls' age at marriage can minimize adolescents' IPPV and other health risks associated with adolescent childbearing.

## Data availability statement

Publicly available datasets were analyzed in this study. This data can be found here: https://dataverse.unc.edu/dataset.xhtml;jsessionid=2041ade5046836520c1117daa52b?persistentId=doi%3A10.15139%2FS3%2FDVEI9A&version=&q=&fileTypeGroupFacet=%22Tabular+Data%22&fileAccess=&fileTag=&fileSortField=&fileSortOrder=.

## Ethics statement

The studies involving human participants were reviewed and approved by icddr,b ERC. Written informed consent to participate in this study was provided by the participants' legal guardian/next of kin.

## Author contributions

MR and KJ conceptualized the study and designed the analysis. NC and MMH performed the statistical analysis. MR drafted the manuscript. SK provided support in literature review and in writing part of the discussion. KJ, MMH, QN, and SK reviewed the manuscript. All authors reviewed, contributed to finalization of the manuscript, and approved the final version of the article.
